# Varicella zoster virus anterior uveitis complicated by thalamic stroke

**DOI:** 10.1186/s12348-021-00243-5

**Published:** 2021-05-01

**Authors:** Casey L. Anthony, Anastasios P. Costarides, Steven Yeh, Jessica G. Shantha

**Affiliations:** 1 Department of Ophthalmology, Emory University School of Medicine, Atlanta, GA 30322 USA; 2 Truhlsen Eye Institute, University of Nebraska Medical Center College of Medicine, Omaha, NE 68105 USA

## Abstract

We report a case of varicella zoster virus (VZV)-associated anterior uveitis in a patient with weight loss, arthritis and signs of inflammatory bowel disease. Her clinical course included the development of a thalamic stroke secondary to VZV cerebral vasculopathy. Following antiviral therapy, the patient’s neurologic symptoms recovered and her ophthalmic findings improved.

## Case report

A 30-year-old woman with a history of an unintentional, 20-pound weight loss within 1 year, gastrointestinal symptoms suggestive of inflammatory bowel disease, and an inflammatory arthritis had been started on an oral prednisone taper 2 months prior to presentation to our service, starting at a dosage of 25 mg per day. Two weeks after starting prednisone, she developed progressive pain and decreased vision in the right eye. She was diagnosed with a unilateral anterior uveitis, which was initially deemed to be associated with her systemic inflammatory condition by an outside provider. The patient was started on topical prednisolone acetate 1% drops four times daily, with only mild improvement in her symptoms. She was then advanced to difluprednate (Durezol) four times daily, given its increased corticosteroid potency. Because of increasing eye pain and inflammation, she was referred for further management.

**Fig. 1:**
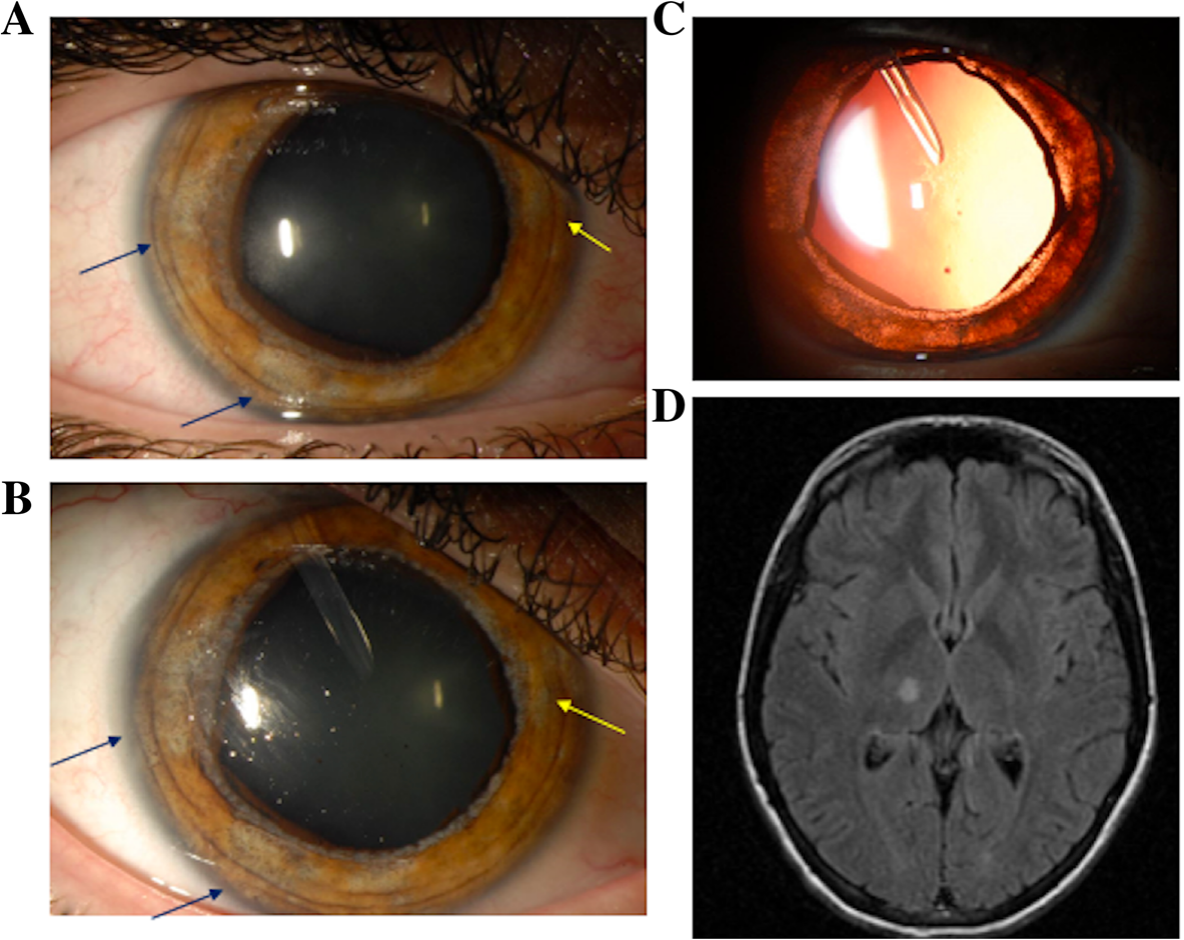
Slit-lamp photo showed multifocal patches of iris atrophy temporally (blue arrows) and nasally (yellow arrow) at presentation (**a**). The areas of atrophy enlarged with increased pigment deposition at follow-up (**b**). These areas are readily visualized as diffuse transillumination defects with retroillumination (i.e. orange/red reflex off the retinal surface) which is visualized through the atrophic iris stroma and epithelium (**c**). A T2 FLAIR magnetic resonance image after the patient’s stroke showed lacunar infarcts within the right thalamus (**d**)

The patient’s presenting Snellen visual acuity was 20/40 in the right eye (OD) and 20/20 in the left eye (OS). The pupils were 9 mm and akinetic OD and 5 mm with brisk light reactivity OS. Her intraocular pressure (IOP) was extremely elevated at 65 mmHg OD (Normal 9–22 mmHg) and 21 mmHg OS. Slit lamp examination showed pigmented, granulomatous keratic precipitates (KP) within the inferior cornea, 3+ anterior chamber cell, diffuse iris atrophy with pigment on the zonular fibers of the lens, and ectropion uveae (Fig. 1). Gonioscopy showed an open angle to the ciliary body band 360 degrees with 3+ pigmented cells within the inferior angle OD. Dilated fundus exam was unremarkable in both eyes. The cup-to-disc ratio was 0.1 in the right eye and 0.2 in left eye with no evidence of glaucomatous optic neuropathy. Mild optic disc hyperemia was observed in the right eye. At the time of presentation, she had been off oral prednisone for 3 weeks and off topical corticosteroid drops for 3 days.

A diagnostic anterior chamber paracentesis to assess the aqueous humor was positive for VZV DNA by PCR testing and negative for HSV, CMV, and toxoplasmosis DNA. Serologic testing showed positive VZV IgG antibody. She was diagnosed with VZV-associated hypertensive anterior uveitis, prompting the initiation of oral acetazolamide and ophthalmic timolol, dorzolamide, and brimonidine for ocular hypertension, as well as valacyclovir 1 g TID and prednisolone acetate 1% every 2 h to treat the VZV-associated anterior uveitis.

Two days after starting valacyclovir, the patient developed left-sided lip, arm, and leg numbness and tingling and presented to an outside hospital for a cerebrovascular accident. MRI showed a right-sided thalamic lacunar infarct (Fig. 1). Additional workup included a carotid ultrasound and transthoracic echocardiogram, which were unremarkable. A lumbar puncture was discussed with the patient but ultimately deferred given that the patient had received antiviral therapy prior to presentation. The thalamic stroke was thought to be due to VZV-associated CNS vasculopathy, and a two-week course of IV acyclovir was administered with resolution of the majority of her CNS symptoms. She resumed oral valacyclovir and topical corticosteroid after her IV acyclovir treatment. The patient’s eye pain improved and her visual acuity remained stable with an IOP of 13 mmHg OD 2 weeks after her hospitalization.

However, 2 months after her stroke, she presented with a recurrence of pain and blurred vision OD while still on valacyclovir 1 g TID. The patient’s visual acuity had declined to 20/100 and her IOP was elevated at 39 mmHg. Anterior segment exam showed 1+ ciliary injection and 3+ anterior chamber cell. She was switched from prednisolone to difluprednate with minimal improvement in the intraocular inflammation. Because of her persistently elevated IOP, she underwent placement of an Ahmed tube shunt with a postoperative improvement of her IOP to 11 mmHg. The patient’s ocular inflammation eventually resolved while she remained on valacyclovir (1 g TID) and topical prednisolone acetate 1% QID.

At 6-month follow-up, the patient’s symptoms and exam findings improved with a visual acuity of 20/80 and IOP of 15 mmHg, but she continued to exhibit 1+ pigmented cell. At 10 months following her initial evaluation, her exam stabilized with visual acuity of 20/50 and only rare anterior chamber pigment cells (Figure). The patient has continued prednisolone acetate 1% TID and timolol BID. The valacyclovir dosage was reduced to 1 g BID at her 12-month follow-up visit where her uveitis remained inactive and there were no residual neurologic defects from the CNS vasculopathy. She was also diagnosed with early Crohn’s disease during her clinical course, but no additional systemic immunosuppressive medications were recommended at the time given the risk of exacerbating the viral disease process.

## Discussion

Anterior uveitis can be associated with infectious or noninfectious etiologies, and timely diagnosis is essential to guide appropriate treatment and minimize the potential for systemic morbidity, particularly in cases where non-ophthalmic findings develop [1, 2]. Our patient was undergoing workup for inflammatory bowel disease and arthritis and was treated initially with oral corticosteroids, which may have contributed her protracted disease course. Anterior uveitis is deemed to be idiopathic in approximately 50% of cases and often does not require extensive workup, particularly for the first episode of anterior uveitis; however, a viral etiology (i.e. varicella zoster, herpes simplex, cytomegalovirus, or rubella) should be strongly considered in the differential diagnosis when uveitis is accompanied by ocular hypertension or iris atrophy, both of which were observed in our patient’s case [3].

VZV-associated anterior uveitis varies in spectrum and appropriate antiviral therapy with corticosteroid may lead to disease resolution in some patients following its acute presentation. However, in other patients, chronic or recurrent disease may develop and require long-term prophylactic antiviral therapy. Complications can include iris atrophy, pupillary abnormalities (i.e. posterior synechiae, pupillary dysmotility from sphincter dysfunction and atrophy), acute IOP elevation, and glaucomatous optic neuropathy. Because of the diffuse and severe iris atrophy, we deemed the mydriasis in our patient to have occurred due to iris sphincter damage. A thalamic stroke extending to the brainstem may also lead to pupillary abnormalities; however, no evidence of brainstem ischemia was observed and the pupillary abnormalities were consistent with the local iris atrophic changes observed. Interestingly, our patient did not show typical cutaneous manifestations and presented at a younger age than most individuals, as VZV anterior uveitis is more commonly observed in elderly patients. Even in the absence of a history of herpetic zoster ophthalmicus, clinical suspicion for a viral process should remain high in patients with characteristic ophthalmic findings including hypertensive anterior uveitis, particularly in conjunction with sectoral iris atrophy [1]. PCR of aqueous humor is a highly sensitive test to confirm the diagnosis following aspiration of intraocular fluid, which can be performed in the ophthalmic clinic setting [4].

After presenting with anterior uveitis, the patient developed a stroke, which was consistent with CNS vasculopathy previously described with VZV. Prior studies show an up to 10-fold increased stroke risk in patients with VZV as a result of viral infection in the arterial wall of cerebral vasculature. VZV vasculopathy was historically associated with large-vessel stroke and acute hemiplegia, but the clinical spectrum of VZV vasculopathies can include transient ischemic attacks, ischemic and hemorrhagic stroke involving both small and large vessels, aneurysm, cranial neuropathies, and venous sinus thrombosis. While VZV vasculopathy is treatable, this diagnosis is often missed and no antiviral treatment is administered, as one-third of patients have no documented history of a zosteriform rash, and many patients may have normal CSF findings [4–7]. It is notable that the patient developed CNS vasculopathy despite valacyclovir therapy. Immunohistochemical studies have shown VZV antigen within the adventitial layer of arteries early during infection. Downstream mechanisms in the pathogenesis of vasculopathy include disruption of internal elastic lamina, thickening intima composed of myofibroblasts, potentially contributing to luminal occlusion, and a paucity of smooth muscle cells with subsequent loss of vessel integrity [8]. CD4+ and CD8+ T-cells, CD68+ macrophages, and CD20+ B-cells have also been observed in the adventitia and intima, indicating a role for inflammatory pathways in this disease process. Thus, while appropriate antiviral therapy is paramount to limit viral replication, a number of other disease processes may have contributed to the patient’s unusual disease course [9].

The differential diagnosis of the combination of anterior uveitis and stroke includes both infectious and noninfectious conditions. These include viral etiologies (VZV, HSV), tuberculosis, syphilis, sarcoidosis, granulomatous polyangiitis, and neuro-Behcet’s disease. Systemic lupus erythematosus and rheumatoid arthritis are less commonly associated with anterior uveitis, and more often associated with scleritis as their predominant ocular inflammatory disease finding, but still warrant consideration in patients with typical systemic findings. Our patient’s treatment of her stroke included empiric intravenous acyclovir by the treating facility given her recent uveitis diagnosis. Ultimately, the patient’s neurologic symptoms and disease findings abated.

The initial diagnosis of VZV-associated hypertensive anterior uveitis, diffuse iris atrophy with pigment release, and disease course was atypical in the patient’s age, degree of iris pigment change, and the potentially debilitating stroke. Few cases have been reported of a stroke following diagnosis of ocular VZV [10, 11]. However, recognition of the relationship between the ophthalmic and CNS manifestations was important for delivery of multidisciplinary care. In addition, this case emphasizes the importance of differentiating between infectious and noninfectious etiologies of uveitis, as there were additional concerns about VZV persistence and potential disease recurrence in the eye with corticosteroid in the absence of appropriate antiviral or other immunosuppressive medications (i.e. disease-modifying anti-rheumatic drugs targeting uveitis associated with collagen vascular disease). Besides the potential of exacerbating anterior uveitis with immunosuppression, individuals with viral anterior uveitis may also develop sight-threatening retinitis (i.e. acute retinal necrosis) in the setting of delayed diagnoses leading to a resultant delay in antiviral therapy [12]. In this report, an 87-year-old male patient presented with unilateral anterior uveitis initially treated with topical corticosteroids alone. Two weeks later, a central retinal artery occlusion was observed and VZV-ARN was subsequently diagnosed, prompting antiviral therapy. Unfortunately, the visual acuity had declined to counting fingers without significant recovery at 6-months follow-up [12]. The recognition of ocular VZV was instrumental for prompt antiviral therapy and identifying the underlying cause of stroke in our patient.

## Data Availability

Data sharing is not applicable to this article as no datasets were generated or analyzed during the current study.

## Abbreviations

BID: two times per day
CNS: central nervous system
HSV: herpes simplex virus
IOP: intraocular pressure
MRI: magnetic resonance imaging
OD: right eye
OS: left eye
PCR: polymerase chain reaction
QID: four times per day
TID: three times per day
VZV: varicella zoster virus
